# Conditional gene expression systems in the transgenic rat brain

**DOI:** 10.1186/1741-7007-10-77

**Published:** 2012-09-03

**Authors:** Kai Schönig, Tillmann Weber, Ariana Frömmig, Lena Wendler, Brigitte Pesold, Dominik Djandji, Hermann Bujard, Dusan Bartsch

**Affiliations:** 1 Department of Molecular Biology, Central Institute of Mental Health and Heidelberg University, Medical Faculty Mannheim, J5, 68159 Mannheim, Germany; 2 Department of Addictive Behavior and Addiction Medicine, Central Institute of Mental Health, Heidelberg University, J5, 68159 Mannheim, Germany; 3 German Cancer Research Center (DKFZ), Molecular Immunology, Im Neuenheimer Feld 580, 69120 Heidelberg, Germany; 4 Zentrum für Molekulare Biologie Heidelberg, Im Neuenheimer Feld 282, 69120 Heidelberg, Germany

**Keywords:** CaMKIIα, conditional expression, Cre/loxP, rat model, Tet system, transgenic rat, tTA

## Abstract

**Background:**

Turning gene expression on and off at will is one of the most powerful tools for the study of gene function *in vivo*. While several conditional systems were successful in invertebrates, in mice the Cre/loxP recombination system and the tet-controlled transcription activation system are predominant. Both expression systems allow for spatial and temporal control of gene activities, and, in the case of tet regulation, even for the reversible activation/inactivation of gene expression. Although the rat is the principal experimental model in biomedical research, in particular in studies of neuroscience, conditional rat transgenic systems are exceptionally rare in this species.

**Results:**

We addressed this lack of technology, and established and thoroughly characterized CreERT2 and tTA transgenic rats with forebrain-specific transgene expression, controlled by the CaMKII alpha promoter. In addition, we developed new universal rat reporter lines for both transcription control systems and established inducible and efficient reporter gene expression in forebrain neurons.

**Conclusions:**

We demonstrate that conditional genetic manipulations in the rat brain are both feasible and practicable and outline advantages and limitations of the Tet and Cre/loxP system in the rat brain.

## Background

The genetic manipulation of target genes in transgenic animals *in vivo *is one of the most valuable tools for assessing gene functions and for modelling human diseases. The most popular approaches for the generation of genetically modified mice are the targeted engineering of genomic DNA in embryonic stem cells to introduce gene knockouts and the DNA microinjection into the pronuclei of fertilized eggs, which results in random integration of the transgene into the genome for the overexpression of gene products.

These valuable techniques have inherent limitations related to the irreversibility and ubiquity of the germ line genetic modifications. If essential genes are targeted, this will potentially lead to embryonic lethality or activation of compensatory mechanisms, which complicates or even impedes the phenotypic analyses of these animal models [1]. In particular this holds true for the large number of genes involved in the formation of neuronal structures emerging late in embryonic development or at early postnatal stages. To overcome these limitations, several techniques for the spatial and temporal control of gene expression in genetically modified mice have been proposed and developed [2], of which the Cre/loxP recombination system [3] and the tet-controlled transcription activation system (Tet system [4]) have become the most widely applied and characterized [1].

Cre is a site-specific recombinase that catalyses recombination between its recognition sites, loxP, leading to an inversion or deletion of a loxP-flanked DNA sequence. Hence, Cre recombinase can be applied to delete genes or to activate transcription by removing a transcriptional termination (STOP) sequence [1]. To obtain an inducible version of Cre, the recombinase was fused to a mutant form of the human oestrogen receptor, leading to cytoplasmic localization and therefore inactivation of the fusion protein (CreERT2) [5]. Once the ligand tamoxifen is applied, CreERT2 translocates into the nucleus, where recombination takes place.

The Tet system is based on two central elements, the tetracycline (tet)-controlled transactivator (tTA) and a specific responsive promoter (Ptet), which controls expression of the transgene [4]. Ptet is specifically activated by binding of tTA. Tet and tet derivatives such as doxycycline hydrochloride (Dox) interfere with the DNA-binding activity of tTA, thereby abolishing transcriptional activation of Ptet. By tissue-specific expression of Cre recombinase or tTA, the impact of transgenic perturbation can be limited to defined cell populations.

For many scientific questions the rat is the preferential animal model [6]. This is mainly related to its larger body size, relevance to human physiology and large body of experimental experience with these animals [7]. Rats are of particular importance in neuroscience because they are the preferred species for behavioural testing of higher cognitive functions, multielectrode recordings, studies of neuronal regeneration [8] and for gene therapy experiments [9].

Up to now, the establishment of techniques for the conditional manipulation of genes in the rat is far behind those for mice [10]. Furthermore, in light of the recent technological breakthroughs that allow targeted genomic manipulations in rats, including the application of zinc finger or transcription activator-like effector nucleases [11-13] and the development of germ line-competent rat embryonic stem cells [14,15], there is an urgent need to introduce conditional technologies for rats and to develop specific Cre driver lines to realize tissue-specific genetic modifications.

In this report, we describe the generation of transgenic rat lines expressing tTA and the tamoxifen-inducible Cre recombinase CreERT2 under the control of the forebrain-specific Ca^2+^/calmodulin-dependent protein kinase IIa (CaMKIIα) promoter. For functional characterization of the CaMKIIα-tTA and CaMKIIα-CreERT2 lines, we generated novel tTA and Cre reporter lines that allow faithful monitoring of spatial and temporal Ptet-controlled and Cre/loxP-mediated gene regulation. These novel tTA and CreERT2 lines can be used for inducible transgene overexpression, as exemplified herein with reporter genes, or for inducible gene knockouts once loxP-flanked gene alleles become available for the rat genome.

## Results and discussion

### Inducible gene expression in rats using the Tet system

**Figure 1:**
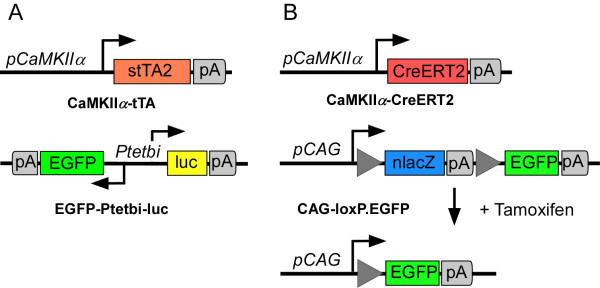
**Conditional expression systems in the rat brain**. **(A) **Tet regulatory system. Schematic outline of the constructs used to generate the transgenic rat lines CaMKIIa-tTA and EGF-Ptetbi-luc rats. The CaMKIIa promoter fragment is used to drive expression of the transcriptional activator tTA, which binds in the absence of doxycycline (Dox) - but not in its presence - to the tetO containing bidirectional minimal promoter Ptetbi. tTA binding activates Ptetbi and leads to the simultaneous expression of the reporter genes EGFP and luciferase (luc). **(B) **CreERT2 inducible gene expression system. Schematic outline of the transcription units incorporated in the CaMKIIa-CreERT2 and CAG.loxP.EGFP rat lines. In double transgenic CaMKIIa-CreERT2/CAG.loxP.EGFP rats, the CaMKIIa promoter fragment controls expression of the tamoxifen-inducible Cre recombinase CreERT2. The Cre reporter transgene is based on a CAG promoter controlled transcriptional unit, in which the lacZ gene (nlacZ, with nuclear localization signal) serves as a STOP fragment to prevent transcription of the posterior gene EGFP. In the non-recombined configuration, lacZ is broadly expressed but replaced by EGFP when Cre-mediated recombination deletes the loxP-flanked STOP fragment. Dox: doxycycline hydrochloride; EGFP: enhanced green fluorescent protein; luc: luciferase; tTA: tetracycline-controlled transactivator.

To establish the tet-controlled conditional gene expression system in transgenic rats (Figure 1A), we generated two separate Sprague-Dawley (SD) rat strains either carrying the tTA expression unit (referred to as driver line) or the gene of interest under control of the tet-inducible promoter Ptet (referred to as response line). Double transgenic rats were generated by breeding the response lines with driver lines, generating animals heterozygous for both transgenic alleles. Such animals will express the transgene depending on administration of the tet derivative Dox, which can be supplied in the drinking water.

#### Generation of CaMKIIα-tTA rat lines

In mice, an 8.5 kb CaMKIIα 5' regulatory sequence has been shown to efficiently drive transgenic tTA expression in excitatory forebrain neurons [16]. The resulting mouse line (Tg(Camk2a-tTA)1Mmay) is one of the most frequently used transactivator mouse lines generated so far and its application has been a seminal contribution to the field of learning and memory [17]. A similar transgenic rat line would enable and facilitate the study of more complex cognitive tasks. As a similar well-characterized rat promoter fragment was not available and the cross-species use of promoters to express tet controlled transactivators between rats and mice has been successful in the past [18-20], we decided to use the functionally proven mouse CaMKIIα promoter to spatially control tTA expression in transgenic rats (Figure 1A). After DNA microinjection, nine transgenic CaMKIIα-tTA founders could be identified, three of which transmitted the transgene to the F1 generation (lines 4.5, 4.7 and 5.1).

#### Generation of Ptet-controlled rat reporter lines

For functional characterization of CaMKIIα-tTA driver lines, we generated response lines carrying the reporter genes luciferase (*luc*) and enhanced green fluorescent protein (*EGFP*) under the control of the bidirectional tet-inducible promoter (Ptetbi, [21]) (EGFP-Ptetbi-luc line; Figure 1A). The experimental strategy for creating the EGFP-Ptetbi-luc lines has previously been described [22]. In brief, a 75 kb genomic mouse fragment containing the Ptetbi-controlled transcription unit was used for microinjection into fertilized SD rat oocytes. In mice, these genomic sequences have been shown to minimize the influence of the genomic integration site on tet-regulated promoters [22,23]. Two transgenic founders harbouring the full-length transgene could be identified. Both lines showed broad range Dox-dependent Ptet-controlled regulation of luciferase in primary fibroblast cultures after transfection with tTA-encoding plasmids (data not shown). Therefore, both lines were used to establish independent reporter lines (EGFP-Ptetbi-luc 66.1 and 71.1).

#### Analysis of tet-regulated gene expression in double transgenic CaMKIIα-tTA/EGFP-Ptetbi-luc rats

All three transgenic CaMKIIα-tTA driver lines were crossed to the response lines to generate double transgenic CaMKIIα-tTA/EGFP-Ptetbi-luc rats. In these rats, preliminary luciferase luminescence analysis of crude forebrain lysates demonstrated functional Ptet-controlled luciferase expression in two CaMKIIα-tTA driver lines (lines 4.5 and 4.7). These were further used for in-depth analysis of Ptet-controlled gene expression.

**Figure 2:**
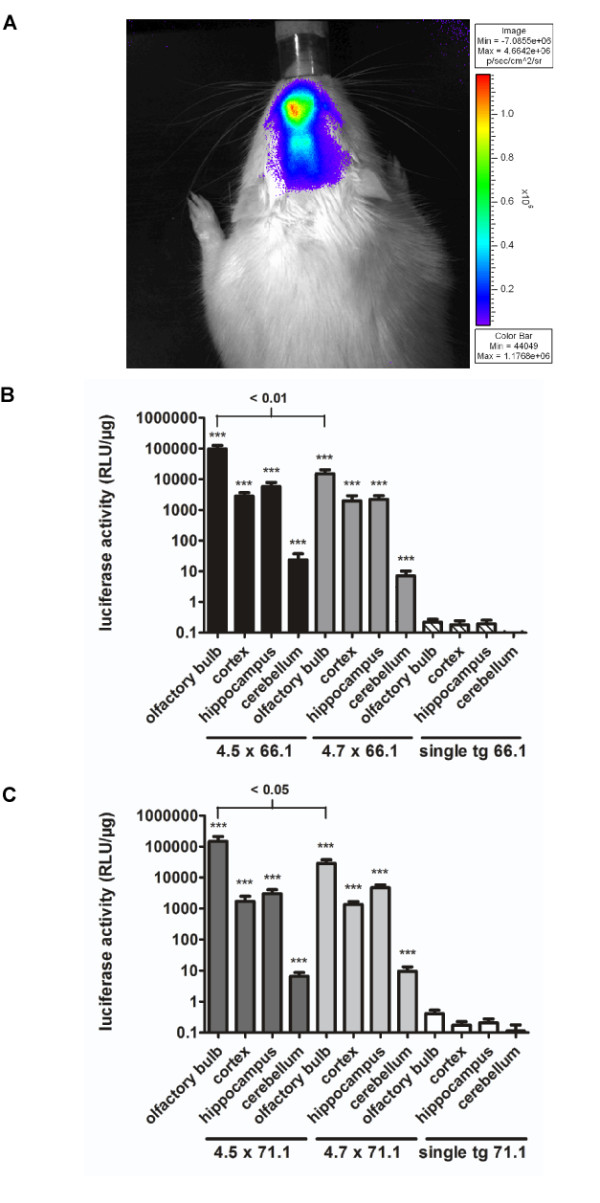
**Luciferase expression in double transgenic CaMKIIα-tTA/EGFP-Ptetbi-luc rats**. **(A) ***In vivo *imaging of luciferase gene activity. Representative example of brain bioluminescence after injection of the luciferase substrate D-luciferin. The rats were anaesthetized during the imaging process using 2.5% isoflurane. Shortly before the measurement, the fur was shaved on top of the head to facilitate the external detection of internally generated photons. Luciferase bioluminescence was restricted to the head region, which demonstrates brain-specific expression in CaMKIIα-tTA/EGFP-Ptetbi-luc rats. Photons were scattered by the skull. **(B,C) **Quantification of luciferase activity in different brain regions. Animals of the CaMKIIα-tTA lines 4.5 and 4.7 were crossed to the reporter lines EGFP-Ptetbi-luc 66.1 (B) and 71.1 (C), respectively, to obtain double transgenic animals of all four combinations. Luciferase activity in lysates of double transgenic and single transgenic rats (line 66.1 and 71.1) is reported as relative light units (RLU) per μg protein and presented on a log scale. Luciferase activity conveyed by both CaMKIIα-tTA lines 4.5 and 4.7 in combination with the Ptet-reporter 66.1 (B) was significantly higher (*P *< 0.001 for all measured brain regions) than single transgenic reporter activity (olfactory bulb: F (2,15) = 304, *P *≤ 0.001; cortex: F (2,14) = 118, *P *≤ 0.001; hippocampus: F (2,14) = 184, *P *≤ 0.001; cerebellum: F (2,14) = 37.3, *P *≤ 0.001). Identical highly significant results (*P *< 0.001 for all subregions versus single transgenic reporter rats of line 71.1) were also found for both tTA driver lines in combination with line EGFP-Ptetbi-luc 71.1 (olfactory bulb: F (2,17) = 174, *P *≤ 0.001; cortex: F (2,17) = 155, *P *≤ 0.001; hippocampus: F (2,16) = 205, *P *≤ 0.001; cerebellum: F (2,16) = 28.3, *P *≤ 0.001) (C). When the luciferase activity mediated by the two transgenic tTA driver lines was compared between the two, line 4.5 showed a significantly stronger luciferase activity in the olfactory bulb (dF = 12, *P *≤ 0.01 in combination with reporter 66.1 in (B); dF = 13, *P *≤ 0.05 with reporter 71.1 in (C)), whereas no significant differences were found in the other brain regions (B,C). Moreover, luciferase activity of Ptet-reporter lines 66.1 and 71.1 with both tTA driver lines (4.5/66.1 versus 4.5/77.1 and 4.7/66.1 versus 4.7/71.1) was not significantly different (B,C). All data are presented as mean values + standard error of the mean. Logarithmic data transformation was performed prior to statistical analysis. Stars represent *P*-values obtained by one-way analysis of variance followed by Bonferroni post hoc test: ***P < 0.001. RLU: relative light units; tTA: tetracycline-controlled transactivator

Transgenic animals of both tTA driver lines were mated with both EGFP-Ptetbi-luc response lines (66.1 and 71.1) to yield double transgenic CaMKIIα-tTA/EGFP-Ptetbi-luc offspring of all four combinations. Individual animals were first studied by non-invasive luciferase imaging [24] to investigate the overall distribution of Ptet-controlled reporter gene expression (Figure 2A). For that purpose, rats were injected intraperitoneally with the luciferase substrate D-luciferin and luciferase activity was monitored noninvasively in anesthetized animals using a photon imaging system [25]. As expected, brain-specific luciferase bioluminescence could be detected only over the head region of CaMKIIα-tTA/EGFP-Ptetbi-luc rats. The observed brain-specific transgenic expression was further confirmed by luciferase measurements in tissue lysates of liver, kidney, heart, lung, spleen and muscle where the enzyme activity did not exceed 1 relative light units (RLU)/μg (data not shown).

To investigate the spatial and quantitative expression pattern in the brain, we prepared tissue lysates of the olfactory bulb, cortex, hippocampus and cerebellum from double transgenic and single transgenic animals and measured luciferase activity (Figure 2B,C). All four strain combinations showed comparably high expression levels in forebrain regions while single transgenic reporter rats of line 66.1 or 71.1 showed practically no luciferase activity, confirming that Ptet-controlled luciferase expression is dependent on tTA-mediated promoter activation.

According to the endogenous CaMKIIα expression pattern, high luciferase activity was found in forebrain regions, that is, in the olfactory bulb, hippocampus and cortex, whereas very low enzyme activity was found in the cerebellum. When luciferase reporter expression was analysed for both reporters (Figure 2B,C), CaMKIIα-tTA line 4.5 showed better expression levels in the olfactory bulb compared with line 4.7 (66.1: *P *≤ 0.01; 71.1: *P *≤ 0.05), whereas luciferase activity in the other regions was comparable. No statistically significant differences were found between the two EGFP-Ptetbi-luc lines used, confirming that both rat lines show equal efficacy in reporting Ptet-controlled gene activation. However, the mean of luciferase expression was slightly higher in the cortex and hippocampus of combination 4.5. × 66.1 compared with all other combinations (Figure 2B,C). As a consequence, we decided to further investigate Ptet-controlled gene expression using double transgenic animals of strain combination 4.5. × 66.1.

**Figure 3:**
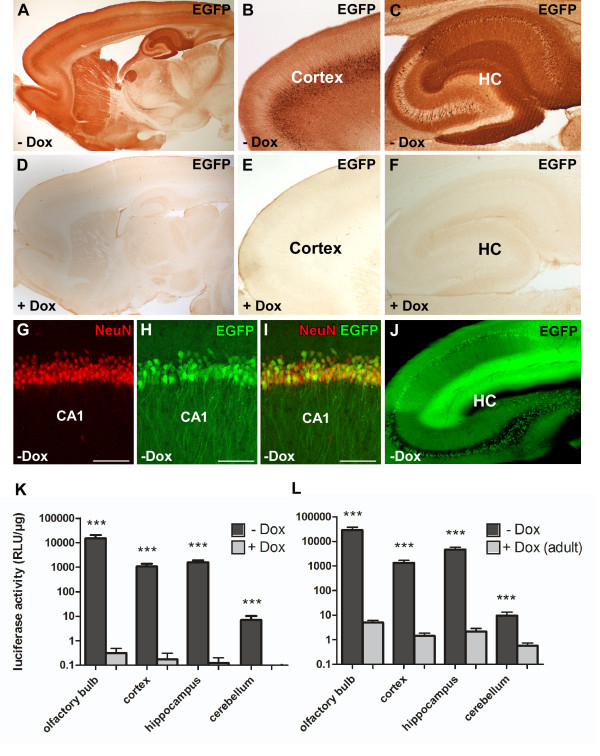
**Doxycycline-mediated gene regulation in double transgenic CaMKIIα-tTA/EGFP-Ptetbi-Luc rats**. **(A-F) **Doxycycline-controlled EGFP expression in double transgenic CaMKIIα-tTA/EGFP-Ptetbi-luc rats was visualized by immunohistochemistry on sagittal sections using an antibody against EGFP. (A-C) In the absence of Dox, strong but mosaic EGFP expression is found in the cortex and hippocampus (HC). (D-F) In adult rats, EGFP expression could be completely inhibited by chronic Dox treatment (1 mg/mL drinking water) from conception until analyses. **(G-I) **Dual-label fluorescent immunohistochemistry of brain slices with the neuronal marker NeuN and EGFP. (I) Co-localization of EGFP and NeuN was frequently found in the CA1 region of the HC indicating Ptet-controlled reporter gene expression in the absence of Dox. **(J) **CaMKIIα-promoter controlled tTA activity was directly visualized by EGFP fluorescence on sagittal brain sections of double transgenic CaMKIIα-tTA/EGFP-Ptetbi-Luc rats in the HC. Strong expression is mainly found in neurons of the CA1 and CA3 region. **(K,L) **Level of luciferase activity in different brain regions in the absence (-Dox, black) and presence of Dox (+Dox, 1 mg/mL, lightly shaded). The measured double transgenic animals were obtained by crossing the CaMKIIα-tTA line 4.5 to the EGFP-Ptetbi-luc 66.1 reporter line. (K) Animals were treated with Dox from conception throughout life until the day of analyses at the age of two months (+Dox, n = 5). Untreated control animals were measured at the same age (-Dox, n = 8). In Dox-treated CaMKIIα-tTA/EGFP-Ptetbi-luc rats, virtually no luciferase activity could be detected, while strong luciferase activity was found in all forebrain-specific regions (cortex, HC, olfactory bulb) of untreated rats (-Dox), leading to a highly significant difference between Dox-treated and untreated rats (***P ≤ 0.001). **(L) **To assess whether reporter gene expression could be suppressed with Dox in adult rats (+Dox adult), double transgenic animals, which had previously not received Dox, were treated with Dox for a period of three weeks during adulthood (-Dox, n = 10, +Dox adult, n = 6). Administration of Dox during adulthood suppressed luciferase activity, leading to a significant difference compared to untreated rats (***P ≤ 0.001). All data are presented as mean values + standard error of the mean. Logarithmic data transformation was performed prior to statistical analysis. Stars represent *P*-values obtained by unpaired t-test. ***P ≤ 0.001. Light units are normalized to the protein content of the lysates. Scale bar: 100 μm. Dox: doxycycline hydrochloride; EGFP: enhanced green fluorescent protein; HC: hippocampus.

Ptet-controlled gene expression in CaMKIIα-tTA/EGFP-Ptetbi-luc rats was further examined on brain slices, taking advantage of the second Ptet-controlled reporter gene *EGFP*. EGFP was either detected by immunohistochemistry (IHC) using a GFP antibody (Figure 3A-F,H,I) or directly by fluorescent microscopy (Figure 3J). EGFP expression was restricted to the forebrain, with strong EGFP-positive cells in the olfactory bulb (glomerular layer), the cortical somatomotor and visual areas and the hippocampal CA1 to CA3 region (pyramidal layer) (Figure 3A-C). Dual-label fluorescence IHC detecting EGFP and the neuronal marker NeuN confirmed neuronal cell specificity of mosaic EGFP expression in CaMKIIα-tTA/EGFP-Ptetbi-luc rats (Figure 3G-I). Ptet-mediated gene expression was most prominent in the hippocampus, with EGFP expression in 58.9% (±8.9%, n = 4) of NeuN-positive cells in the pyramidal layer of the CA1 region; 42.8% (±6.5%, n = 3) GFP-positive/NeuN-positive cells in the CA3; and 46.2% (±5.9%, n = 4) in the hilus of the dentate gyrus. In the cortex, EGFP expression was highly variable. However, in regions with notable reporter gene expression, such as the somatomotor area (layer 5), up to 38.4% (±6.9%, n = 4) of NeuN cells stained positive for GFP.

The possibility to directly visualize EGFP-expressing cells indicates a very strong expression using the Tet system in these transgenic rats (Figure 3J). Luciferase and EGFP are expressed in the same brain areas, which confirms the feasibility of co-expressing two genes at the same time in rats using the bidirectional tet-promoter Ptetbi [21]. For the most part, the expression pattern found in CaMKIIα-tTA rats mirrors the spatial expression pattern found in CaMKIIα-tTA transgenic mice harbouring a similar CaMKIIα-tTA construct (olfactory bulb, cortex, striatum and hippocampus) [16,26,27]. However, only scarce expression was found in the dentate gyrus of the hippocampus and in the striatum, and expression in the cortex was highly mosaic. Endogenous CaMKIIα immunoreactivity is detected in most, if not all, neurons in the forebrain, but not in glial cells [12]. The mouse CaMKIIα promoter might therefore not have the full expression range in transgenic rats. However, for transgenic mice, it has been repeatedly shown that different Ptet transgenes yield varying patterns of expression in combination with the same CaMKIIα-tTA transgene [16,27,28] and these differences are considered to result from integration site-dependent effects on the expression of the Ptet promoter [29,30]. We believe that similar mechanisms in the rat genome could act on the CaMKIIα-tTA transgene as well, leading to the mosaic-like expression pattern.

#### Doxycycline-mediated control of gene expression

Next, we assessed whether Ptet-controlled gene expression could be suppressed with Dox. To that end, we analysed adult double transgenic CaMKIIα-tTA/EGFP-Ptetbi-luc rats which had chronically received Dox (+Dox) during embryonic and postnatal development until postnatal day (P) 60 (1 mg/mL). IHC for EGFP (Figure 3D-F) demonstrated that Ptet-controlled reporter gene expression could be completely suppressed with Dox. These results were confirmed by luciferase measurements in brain extracts of the aforementioned brain regions (Figure 3K). In addition, we show that luciferase activity in adult CaMKIIα-tTA/EGFP-Ptetbi-luc rats, which previously had not received Dox, could be efficiently suppressed by Dox treatment (+Dox adult) when given only during a short period during adulthood (P90 to P110) (Figure 3L). Dox treatment led to a 1000-fold reduction in luciferase expression in all forebrain-specific regions when compared with untreated animals (-Dox). These findings confirm that Ptet-controlled gene expression can be suppressed at any time during the rat's life.

**Figure 4:**
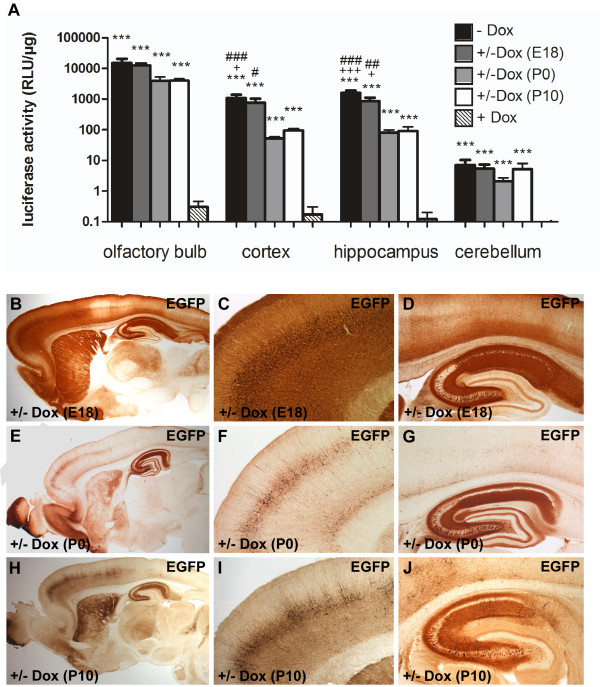
**Activation of reporter gene expression after doxycycline withdrawal**. Double transgenic CaMKIIα-tTA/EGFP-Ptetbi-Luc rats (4.5 × 66.1) were treated with Dox during development until E18, P0 and P10, respectively (10 μg/mL). After 60 days of Dox withdrawal (±Dox), reporter gene expression was analyzed by luciferase measurements of brain regions (A) and EGFP IHC (B-J). **(A) **A highly significant difference in luciferase activity was found between the different groups (olfactory bulb: F (4,22) = 143, *P *< 0.001; cortex: F (4,22) = 79.4, *P *< 0.001; hippocampus: F (4,22) = 125, *P *< 0.001; cerebellum: F (4,22) = 31.5, *P *< 0.001). In forebrain regions (olfactory bulb, cortex, hippocampus), strong luciferase expression was induced by Dox withdrawal at E18, P0 and P10 (±Dox) compared with Dox-treated animals (+Dox). However, in rats which had received Dox until birth (P0) or until P10, luciferase expression did not reach the level of untreated animals (-Dox). This suggests that prolonged Dox treatment during development, in particular after birth, impairs activation of Ptet-controlled gene expression after Dox withdrawal. Data are presented as mean + standard error of the mean. Logarithmic data transformation was performed prior to statistical analysis. Characters (*, +, #) represent *P*-values obtained by one-way analysis of variance followed by Bonferroni post hoc analysis. * represents significance levels versus +Dox (***P ≤ 0.001); ^+ ^versus P10 (^+^*P *≤ 0.05; ^+++^*P *≤ 0.001) and ^# ^versus P0 (^#^*P *≤ 0.05; ^##^*P *≤ 0.01; ^###^*P *≤ 0.001). Light units are normalized to the protein content of the lysates. **(B-J) **IHC confirms and extends the findings of the luciferase assays. Again, Dox-treated rats which had received Dox only until E18 (B-D) showed a strong forebrain-specific reporter protein expression pattern which was comparable to untreated rats (-Dox, Figure 3A-C) whereas the level of expression in all forebrain regions was significantly reduced when Dox was given until P0 (E-G) or P10 (H-J). Dox: doxycycline hydrochloride; E: embryonic day; IHC: immunohistochemistry; P: postnatal day.

If expression of the gene of interest is thought to be detrimental during development or if adaptive changes during development caused by the transgenic overexpression should be avoided, gene expression should be induced only after embryonic development. Hence, we determined whether Ptet-controlled gene expression could be activated when it was previously shut off with Dox (±Dox) for a period of continuous suppression during development. Dox was permanently administered to double transgenic rats during development (10 μg/mL) either until embryonic day (E) 18, P0 or P10, after which Dox was removed from the drinking water. Brain tissue was prepared from double transgenic offspring at P60 for luciferase quantification and IHC analyses (Figure 4). Interestingly, we found an inverse correlation between the length of Dox treatment during development and the efficiency of Ptet-controlled reporter gene expression after Dox withdrawal. Rats treated with Dox until E18 were found to exhibit superior induction of reporter gene activity compared with rats that were treated until P0 or P10 (Figure 4A). However, even in rats treated with Dox until E18 only, the expression level did not fully reach the activation level of untreated animals (-Dox, Figure 4A). GFP antibody staining of rats treated with Dox until E18 revealed inducible gene expression mainly in the olfactory bulb (glomerular layer), the CA1 and CA3 region of the hippocampus and areas of the somatomotor cortex (Figure 4B-D). Rats treated with Dox until P0 and P10 only showed gene activation in the olfactory bulb, the CA3 region and in few cells of the cortex (Figure 4E-J). Previously, a similar weak induction has also been observed in CaMKIIα-tTA mice that were raised with Dox until P0 [26] or P21 [31]. Failure to reach full expression has either been attributed to a Dox clearance problem or to specific epigenetic modifications of the Ptet-promoter sequences in CaMKIIα-positive neurons during the suppression period [26,32,33].

In summary, we demonstrate strong, forebrain-specific reporter gene expression in transgenic rats that can be suppressed with Dox at any desired time. The bidirectional expression module provides a useful tool for the co-regulated expression of two genes at the same time. However, prolonged Dox treatment restricts the ability to reactivate Ptet-controlled gene expression in principle forebrain neurons.

### Inducible gene expression in rats using the Cre/loxP system

The CreERT2/loxP system has been widely used for tissue-specific and inducible gene ablations. Apart from this application, the CreERT2/loxP system can also be used as an alternative to the Tet system for conditional gene overexpression [34,35]. While it does not allow for reversible activation of target genes, it allows for irreversible Cre-mediated deletion or activation of loxP-flanked DNA sequences. We explored this alternative inducible expression system in rats and compared it with the Tet system. For this purpose, rat CaMKIIα-CreERT2 lines were generated and inducible recombination was functionally assessed using a newly generated Cre reporter line.

#### Generation of CaMKIIα-CreERT2 rat lines

Again, the same mouse CaMKIIα promoter fragment was chosen for tissue-specific control of CreERT2 recombinase expression in forebrain neurons [5,36] (Figure 1B). Using pronuclear DNA injections, we were able to generate 14 transgenic CaMKIIα-CreERT2 founders. Offspring from all lines were initially analysed for Cre expression via IHC using a Cre antibody [37], but only eight lines showed notable Cre recombinase expression in the forebrain. We selected four CaMKIIα-CreERT2 lines (327, 396, 404 and 408) which recapitulated the forebrain-specific CaMKIIα expression pattern [38] most adequately with abundant Cre-staining in most parts of the forebrain (Additional file 1), including the olfactory bulb, cortex and hippocampus. Similar to the CaMKIIα-tTA lines described above, the hippocampal dentate gyrus was largely devoid of transgenic expression. Sparse expression was also found in the striatum and thalamus.

#### Generation of Cre reporter rats

We generated a rat Cre reporter line pCAG-loxP.LacZ.loxP-EGFP (CAG-loxP.EGFP) to functionally characterize Cre-mediated recombination (Figure 1B). The establishment of a broadly applicable Cre reporter line critically depends on the widespread expression of its reporter genes, which ideally should not be limited to certain cell types or be influenced by position-effect variegation. Therefore, the chicken β-actin (CAG) promoter, which has been shown to confer rather ubiquitous activity in rodents in nearly all tissues [39-41], was used for the design of the reporter construct. CAG promoter sequences were placed upstream of a loxP-flanked (floxed) STOP cassette containing a nuclear localized synthetic *lacZ *gene and a bovine growth polyadenylation (polyA) signal to ensure transcriptional termination. This *lacZ*/STOP cassette was followed by *EGFP *as a second reporter gene. Under baseline conditions, that is without Cre-mediated recombination, the CAG promoter drives only β-galactosidase (β-gal) but not EGFP expression. Upon Cre-mediated recombination, the *lacZ*/STOP cassette is deleted, which activates CAG-promoter controlled expression of EGFP.

To create transgenic rat Cre reporter lines, the CAG-loxP.EGFP construct was microinjected in fertilized SD oocytes, resulting in 12 Cre reporter founder rats. All founders were first assayed for baseline, non-recombined β-gal activity by X-Gal staining of primary fibroblast cultures derived from ear biopsies (data not shown). The primary fibroblast cultures of four founder animals showed a strong nuclear X-Gal staining and those were selected for further *in vivo *characterization.

Tissue slices and whole organs of single transgenic CAG-loxP.EGFP rats were analysed by X-Gal staining. Line 13 showed the most abundant β-gal signal of all analysed lines (Additional file 2). Here, X-Gal staining was detected in all organs analysed, with strong and broad expression in fibroblasts, lung, kidneys, muscle, heart, spleen, pancreas, stomach and gut. Only in the liver was mosaic expression found.

**Figure 5:**
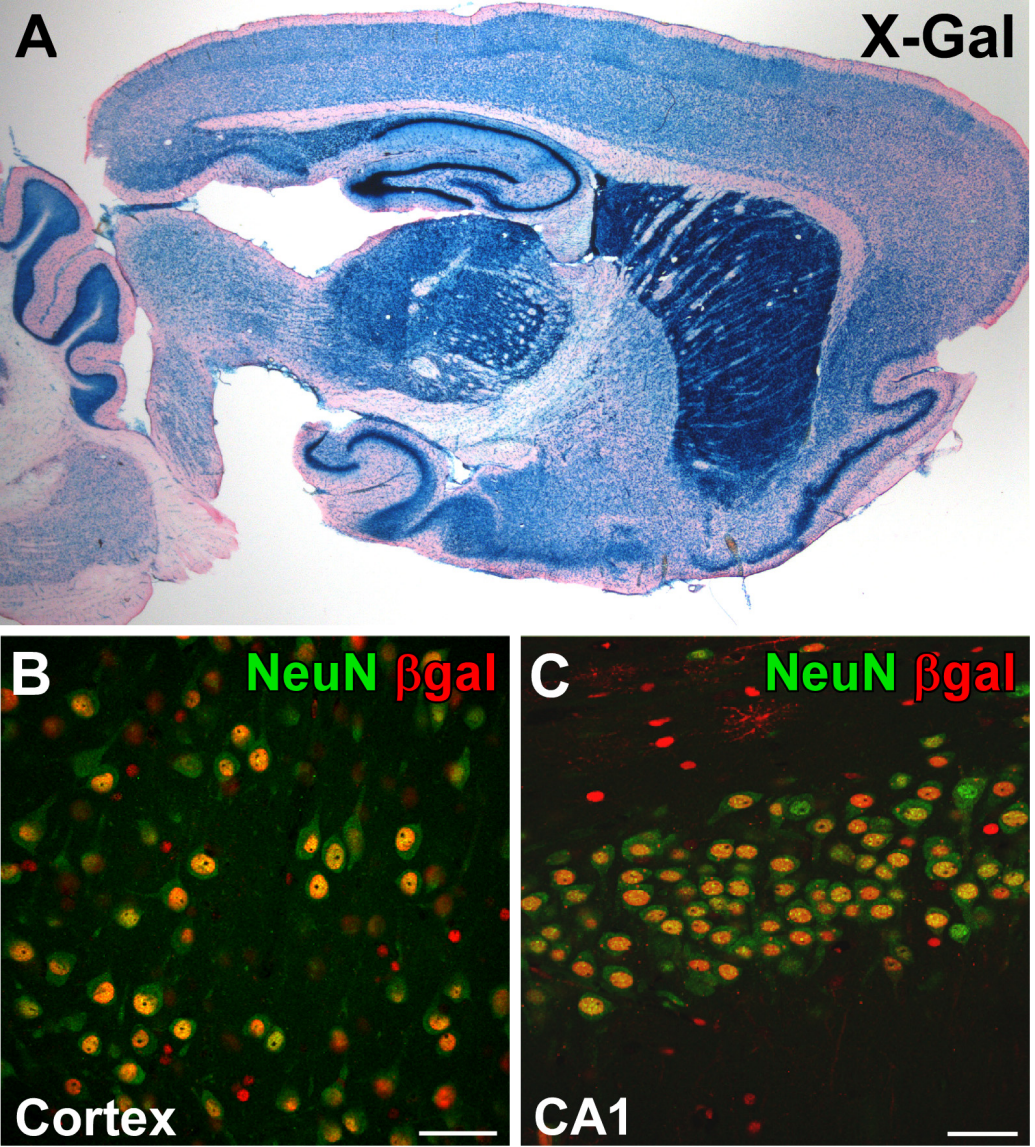
**β-galactosidase expression in the brain of the CAG-loxP.EGFP Cre reporter line 13**. **(A) **X-Gal staining of sagittal brain sections demonstrates widespread β-galactosidase expression throughout the brain. **(B,C) **Neuronal β-galactosidase expression is demonstrated by co-localization of the neuronal marker NeuN and nuclear localized β-galactosidase in the cortex (B) and CA1 region of the hippocampus (C). Scale bar: 50 μm.

Finally, we analysed CAG promoter activity in the brain. Brain sections of transgenic CAG-loxP.EGFP offspring of the four β-gal-positive CAG-loxP.EGFP Cre reporter lines were examined for β-gal expression using X-Gal staining and IHC. Again, X-Gal staining of line 13 showed the strongest and broadest signal in the brain (Figure 5A). Dual-label fluorescent IHC with antibodies against β-gal and NeuN (Figure 5B,C) demonstrated that most NeuN-positive neurons in the brain also stained positive for β-gal (CA1: 93.4% ± 2.1%; cortex 91.8% ± 4.7%; n = 3). By contrast, EGFP expression could not be detected, which proves that the polyA signal downstream of the *lacZ *gene reliably functions as a transcriptional STOP fragment, leading to tight regulation of recombination-dependent EGFP expression. Our results in rats demonstrate the broad applicability of the CAG-loxP.EGFP line 13 for functional characterization of newly generated tissue-specific rat Cre lines.

#### Analysis of Cre-mediated gene expression in double transgenic CaMKIIα-CreERT2/CAG-loxP.EGFP rats

To functionally examine the spatial and temporal pattern of CreERT2 recombinatorial activity, we crossed rats of the four CaMKIIα-CreERT2 lines (327, 396, 408 and 404) to our Cre reporter line CAG-loxP.EGFP to generate bitransgenic CaMKIIα-CreERT2/CAG-loxP.EGFP rats.

**Figure 6:**
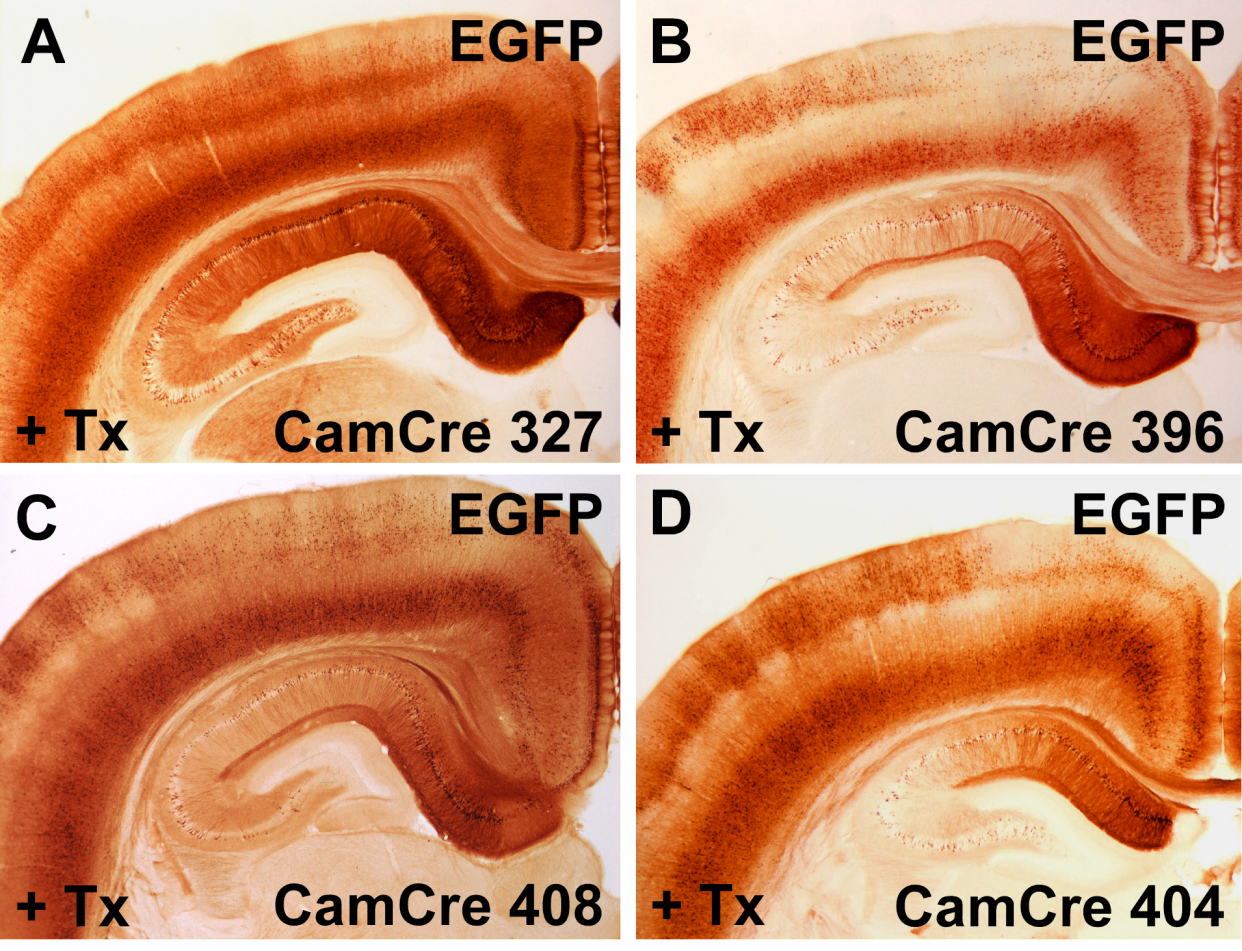
**CaMKIIα-CreERT2-mediated recombination in the rat brain**. Functional recombination using different CaMKIIα-CreERT2 founder lines was assessed with tamoxifen-induced double transgenic CaMKIIα-CreERT2/CAG-loxP.EGFP rats. Double transgenic rats were injected with tamoxifen for five consecutive days. Ten days after the last injection, brain sections were prepared and analyzed by EGFP immunohistochemistry. EGFP expression, which indicates Cre-mediated recombination, is restricted to forebrain structures. Recombination efficiency is dependent on the Cre driver lines used. **(A) **CaMKIIα-CreERT2 line 327, **(B) **line 396, **(C) **line 408 and **(D) **line 404. EGFP: enhanced green fluorescent protein.

**Figure 7:**
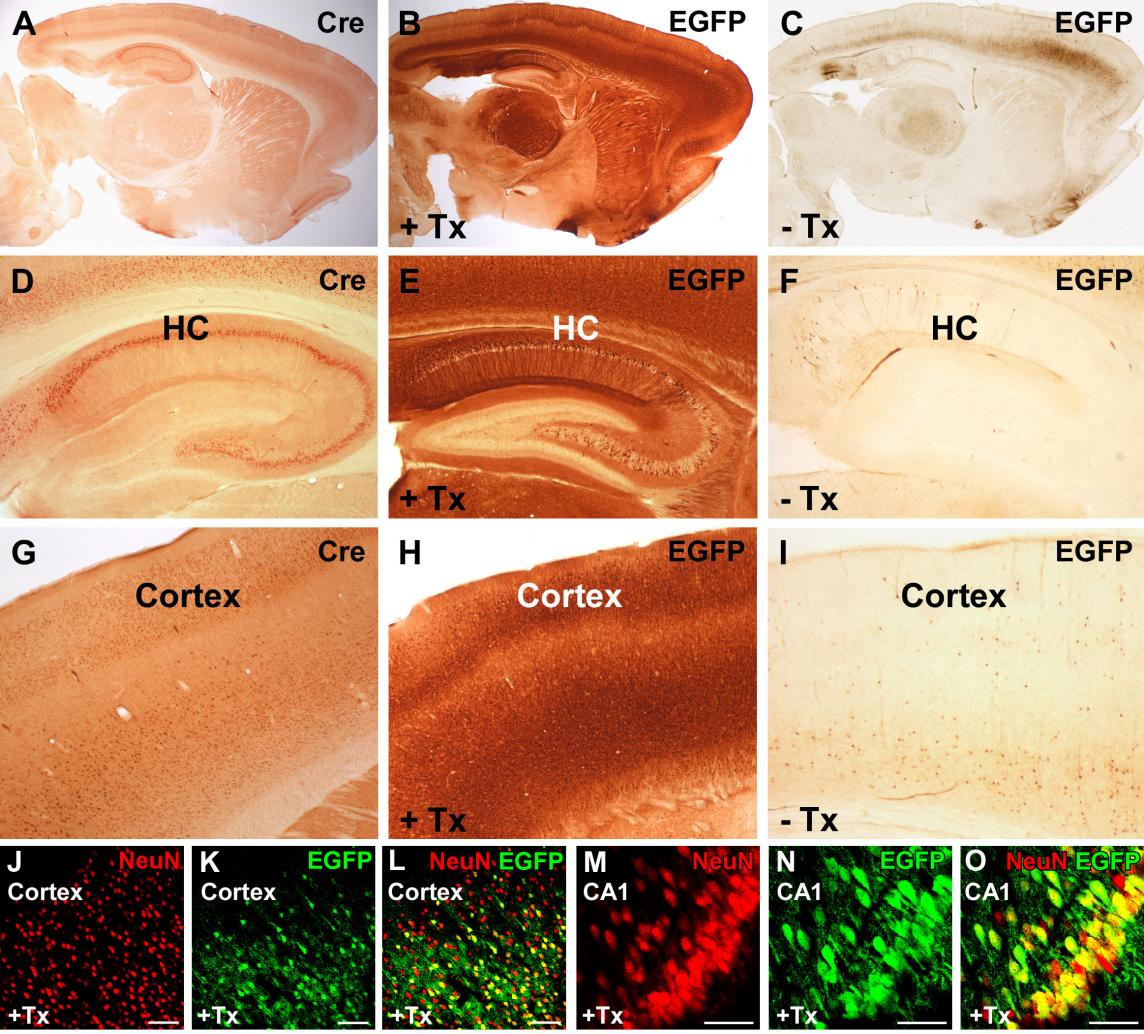
**Tamoxifen-inducible recombination in double transgenic CaMKIIα-CreERT2/CAG-loxP**.EGFP rats. Brain slices were immunostained for **(A,D,G) **Cre recombinase or **(B,C,E,F,H,I) **EGFP expression. Tamoxifen (+Tx)-induced EGFP (B,E,H) expression mirrors Cre expression (A,D,G), whereas uninduced animals (-Tx) only show low background recombination (C,F,I). **(J-O) **Brain slices of tamoxifen-induced CaMKIIα-CreERT2/CAG-loxP.EGFP rats were double-stained for NeuN and EGFP. Co-localization of EGFP and NeuN demonstrated successful Cre-mediated recombination in neurons of the cortex (L) and the CA1 region of the hippocampus (O). Scale bars: J-l 100 μm, M-O 50 μm. EGFP: enhanced green fluorescent protein.

At the age of 10 weeks, double transgenic rats were intraperitoneally injected with tamoxifen (40 mg/kg) alternately once or twice per day, resulting in a total of seven tamoxifen injections over five consecutive days. Ten days after the last tamoxifen injection, animals were analysed with IHC for EGFP to assess forebrain-specific recombination (Figure 6). Of the four different transgenic Cre lines, CaMKIIα-CreERT2 line 327 showed the most efficient forebrain-specific recombination, that is, EGFP expression, in the hippocampus and in the cortex (Figures 6A and 7E,H), whereas only scattered recombination was found in the striatum and thalamus (Figure 7B). Outside the forebrain, EGFP-positive cells were absent (Figure 7B). This forebrain-specific recombination pattern concurs with the recombination pattern found in CaMKIIα-CreERT2 mice, where inducible Cre activity was primarily found in the hippocampus and cortex and only low levels of CreERT2 expression were identified in the striatum and thalamus [42]. Importantly, the recombination pattern (EGFP-positive) closely mirrored Cre expression (Figure 7A,B,D,E,G,H), demonstrating the excellent functionality of the reporter line to detect Cre-mediated recombination in the brain. We assume that the transgenic Cre reporter rat described herein harbours multiple, randomly integrated DNA fragments and might therefore represent an easily accessible genomic locus, ideally suited for a broad and sensitive detection of Cre-mediated recombination.

Next, we evaluated background recombination without tamoxifen treatment (Figure 7C,F,I). Sparse recombined EGFP-positive cells could be identified in various forebrain regions, for example, in the cortical somatomotor area, the postsubiculum and the piriform area. Such tamoxifen-independent recombinase activity has also been described for inducible CreERT transgenic mouse lines [43-45] and is caused by the incomplete trapping of the Cre fusion protein in the cytosol in the absence of tamoxifen. Low Cre activity in absence of the ligand was also found in CaMK-CreERT2 mouse lines [42]. Interestingly, background recombination was not observed in CAG-loxP.EGFP rats mated with less active CreERT2 driver lines, thereby confirming the observation that the ligand-independent activity of inducible Cre lines correlates with its intracellular concentration [46].

To assess cell-type-specific neuronal recombination after tamoxifen induction, double transgenic CaMKIIα-CreERT2/CAG-loxP.EGFP rats were analysed with dual-label fluorescent IHC using antibodies against EGFP and NeuN (Figure 7J-O). Confocal microscopy of hippocampal regions revealed 55.6% (±14.2%, n = 4) recombined EGFP-positive/NeuN-positive neurons in CA1; 50.7% (±4.5%, n = 3) in CA3; 52.7% (±11%, n = 3) in the hilus; and 47.4% (±22.7%, n = 3) in cortical neurons.

In combination with the Cre reporter line CAG-loxP.EGFP, we demonstrate effective temporal regulation of Cre activity by tamoxifen treatment in the newly developed rat line CaMKIIα-CreERT2. In the absence of tamoxifen, only minor recombination occurs, whereas the application of tamoxifen results in widespread recombination within forebrain neurons of the hippocampus and cortex. In total, we were able to target about 50% of NeuN-positive neurons in the aforementioned regions. Similar to the Tet system, we assume that position-effect variegations in the transgenic loci are responsible for the overall variable expression [29,30]. We can exclude insufficient tamoxifen induction as the reason for this mosaic expression pattern, because doubling the injection time and thus tamoxifen dose did not lead to a higher recombination rate. Both the CaMKIIα-tTA and CaMKIIα-CreERT2 lines only sparsely mediate expression in the dentate gyrus or in the striatum. This common phenotype is indicative that the used mouse CaMKIIα promoter fragment lacks regulatory sequences needed for expression specifically in this region in transgenic rats.

To date, only a limited number of Cre-expressing rat lines have been published [47,48]. Tissue specificity and temporal control of recombination was obtained by local injections of Cre-expressing adenoviruses [49,50]. However, an increasing demand for tissue-specific Cre-expressing rat lines can be anticipated in the near future, as the first successful gene targeting in rat embryonic stem cells was recently published [51] and several other tools for the targeted modification of the rat genome, such as zinc finger nucleases [11,12] or transcription activator-like effectors [52], are emerging. Thus, there is little doubt that conditional alleles of target genes will also become available in the rat. Another attractive application is the injection of Cre-dependent viruses to achieve optogenetic control of genetically defined cell types in rats [48]. Moreover, as depicted here with the newly developed Cre reporter line, the Cre/loxP technology can be employed for the tissue-specific overexpression of transgenes or alternatively for the knockdown of endogenous gene activities using polymerase II controlled small hairpin RNAs [53,54]. The presented Cre reporter line CAG-loxP.EGFP should provide a versatile tool for the characterization of newly developed Cre rat lines in both neural and non-neural tissues.

## Conclusions

The laboratory rat was the earliest mammalian species domesticated for scientific research and has been used as such for over 150 years [6]. Although constitutive overexpression of genes in transgenic rats has been successfully applied to generate relevant models for gene-related diseases [55-57], only limited attempts have been made in the past to establish transgenic rat models with inducible and tissue-specific gene expression and no one has successfully addressed the brain [58]. In transgenic mice, the development of conditional strategies to control gene expression in a spatial and temporal manner has been crucial for modelling human diseases and deciphering tissue-specific gene functions [1]. With new emerging technologies, such as the presented forebrain-specific Cre or tTA driver lines, techniques in transgenic rats will close the gap with current mouse technologies. Such rat-based disease models will complement existing mouse models and the comparison of both will enable a better differentiation between rodent-specific and general mammalian phenotypes [59,60].

Our experiences with both inducible systems suggest complementary applications. The Tet system should be applied for the overexpression of transgenes only during embryonic development or until a defined time point during adolescence, which then can be easily turned off by feeding Dox. By contrast, with the CreERT2-based system, a previously inactive expression module is efficiently activated any time after birth.

## Methods

### Generation of transgenic rats

#### EGFP-Ptetbi-luc, CaMKIIα-tTA and CaMKIIα-CreERT2 transgenic lines

The generation of the EGFP-Ptetbi-luc tet-inducible reporter line has been described previously [22]. For the generation of forebrain-specific tTA and Cre-expressing rats, a similar strategy was applied as published by Mayford *et al. *[16]. In brief, the EcoRI-BamHI fragment coding for tTA2s (from pUHT61-1 [61]) and the EcoRI CreERT2 fragment (derived from pCre-ERT2 [5]), respectively, were placed downstream of the 8.5 kb CaMKIIa promoter sequence (pMM403). Previously, the cDNAs had been flanked by artificial introns at the 5' and 3' end (from pNN265 [62]). A SV40 polyA sequence served as the transcriptional termination signal. The CaMKIIα promoter expression cassettes were separated from the vector by digestion with SfiI, purified by DNA extraction from agarose gel and microinjected at a concentration of 2 ng/μL into fertilized SD eggs (Charles River, Sulzfeld, Germany using procedures previously described [63]. All experimental procedures were approved by the Animal Welfare Committee (Regierungspräsidium Karlsruhe) and carried out in accordance with the local Animal Welfare Act and the European Communities Council Directive of 24 November 1986 (86/609/EEC).

#### pCAG-loxP.EGFP Cre reporter line

For monitoring Cre activity in transgenic rats, we designed the expression vector pCAG-loxP-lacZ-loxP.IRES-EGFP, a double-reporter construct, in which the ubiquitous cytomegalovirus enhancer/CAG promoter controls the expression of the *lacZ *gene before and *EGFP *after Cre-mediated recombination. The construct was assembled using the CAG promoter of the plasmid pCAβ, which was cloned upstream of the nuclear localized *nlacZ *gene from pMODlacZ (Invivogen, Toulouse, France) and a bovine growth hormone polyA signal, the latter being flanked by loxP sites. The expression cassette is followed by a multiple cloning site to insert a gene of interest, an internal ribosome entry site (IRES) sequence from the encephalomyocarditis virus (derived from plasmid ETL[64]) and the EGFP open reading frame (Clontech, Saint-Germain-en-Laye, France), followed by a SV40 polyA sequence. For the generation of transgenic rats harbouring the Cre double-reporter construct, a PmeI-NotI fragment was released from the pCAG-loxP-lacZ-loxP.IRES-EGFP vector and microinjected at a concentration of 2 ng/μL into fertilized SD rat eggs. Founder rats and their offspring were analysed by Southern blotting and polymerase chain reaction of tail DNA using primers for Cre, stTA [65], synlacZ (synlacZ_for: 5'-GCTCAGGTCTCTCAATGGAG-3', syn lacZ_rev 5'-CCAGACATCCTCCACATGTC-3') and EGFP (eGFP_for 5'-TTCAAGGACGACGGCAACTACAAG-3', eGFP_rev 5'-CGGCGGCGGTCACGAACTCC-3'). DNA was prepared using the DNEasy Blood & Tissue Kit (QIAGEN, Hilden, Germany).

### Luciferase imaging *in vivo*

Double transgenic CaMKIIα-tTA/EGFP-Ptetbi-luc rats were injected intraperitoneally with an aqueous solution of D-luciferin (150 mg D-luciferin per kilogram of body weight) and immediately placed into a light-tight chamber. Subsequently, bioluminescent imaging was performed following published procedures [24,25]. The emitted light was detected with the IVIS imaging system 100 (Xenogen Corporation, Alameda, California, USA) and analysis was performed using the LivingImage software (version 2.50, Xenogen Corporation).

### Determination of luciferase activity in rat tissues

Tissue samples were homogenized in passive lysis buffer (Promega, Mannheim, Germany) using the mixer-mill Tissue Lyser for 20 seconds at 30 Hz with 3 mm tungsten carbide beads. The homogenate was centrifuged for 5 minutes at 14,000 rpm at 4°C. The supernatants were assayed in 10 mL samples for luciferase activity for 1 second using the Luciferase Reporter Assay system (Promega) according to the manufacturer's instructions, in combination with Wallac Victor 2 multilabel counter (PerkinElmer, Rodgau, Germany). An aliquot of the lysates was used to determine the protein concentration by means of an improved Bradford assay (BioRad, Munich, Germany). Luciferase activities were normalized to micrograms protein (RLU/μg).

### Animal treatment (tamoxifen and doxycycline hydrochloride

Dox was dissolved at a concentration of 1 mg/mL (+Dox experiments) and 10 μg/mL (±Dox experiments) in tap water supplemented with 5% sucrose and supplied to the animals *ad libitum*. Tamoxifen (Sigma-Aldrich, Munich, Germany, T5648) was dissolved in a pH neutral medium-chain triglyceride (Neutralöl, Euro OTC Pharma, Bönen, Germany) at a final concentration of 20 mg/mL. Two- to three-month-old rats were injected intraperitoneally with 40 mg/kg body weight of tamoxifen alternating once or twice per day for five consecutive days (starting with a single injection on day 1). Experimental animals for immunohistochemistry and Cre reporter analysis were analysed 10 days after the last injection.

### Immunohistochemistry and β-galactosidase staining

β-gal activity in transgenic rats was characterized by X-Gal staining and IHC. Dissected brains were postfixed with 4% paraformaldehyde at 4°C for 24 to 48 hours. Brain sections (50 μm) were prepared using a vibratome (Leica, Wetzlar, Germany). Floating sections were processed for IHC using the VECTASTAIN ABC system (Vector Laboratories, Burlingame, California, USA) and diaminobenzidine (Sigma-Aldrich) in combination with polyclonal rabbit anti-Cre (a generous gift from G. Schütz, DKFZ, 1:3000) and polyclonal rabbit anti-GFP (Invitrogen, Darmstadt, Germany 1:1000) antibodies.

Double fluorescence IHC was performed to visualize β-gal and GFP expression in NeuN-positive neurons using the primary antibodies chicken anti-β-gal (Abcam, Cambridge, UK, 1:10,000), mouse monoclonal anti-NeuN (Millipore, Schwalbach, Germany 1:4,000) and polyclonal rabbit anti-GFP (Invitrogen, 1:1,000). Brain sections were permeabilized with Triton X-100 (0.1%) for 30 minutes in 1 × PBS at 4°C, washed with 1 × PBS (three times) and blocked with 10% donkey serum in 1 × PBS for 1 hour. The primary antibodies were added to the blocking solution and incubated at 4°C overnight on an orbital shaker. Next day, following three washings with PBS, the sections were incubated in blocking solution containing the secondary antibodies for 1 hour at room temperature. After final washes with 1 × PBS, sections were mounted in Dako fluorescence mounting medium. Secondary antibodies were AF488 donkey α-mouse (Invitrogen, 1:200) and Cy3 donkey α-chicken (1:1,000). Sagittal vibratome sections were examined using confocal laser-scanning microscopes (Nikon C1Si-CLEM, Nikon Imaging Center, BioQuant, Heidelberg, Germany and Leica TCS SP5, Central Institute of Mental Health, Mannheim, Germany).

For β-gal staining, similar brain sections were incubated with X-Gal staining solution (5 mM EGTA, 2 mM MgCl_2_, 0.01% C_24_H_39_O_4_Na, 0.02% NP-40, 10 mM K_3_(Fe(CN)_6_), 10 mM K_4_(Fe(CN)_6_) and 0.5 mg/mL X-Gal in 1 × PBS) at 37°C for several hours. Finally, the stained slices were counterstained with nuclear fast red (Sigma) and mounted with Eukitt (O. Kindler, Freiburg, Germany).

### Ear fibroblast culture and transfection

Ear fibroblast cultures were prepared from ear biopsies of founder animals using a protocol previously described [66]. Depending on the transgene, the cells were directly examined either by X-Gal staining or transfected with tTA expression plasmids using lipofectamine-2000 (Invitrogen) according to the manufacturer's protocol.

### Statistical analysis

Statistical analyses of luciferase data were performed using either t-test or univariate analysis of variance (ANOVA), followed by Bonferroni post hoc analysis. Respective *F*- and *P*-values were calculated using GraphPad Prism 5.0. All data are presented as mean ± standard error of the mean (SEM). A *P*-value ≤ 0.05 was considered statistically significant. Sagittal slices of adult pCAG-loxP.EGFP, CaMKIIα-tTA/EGFP-Ptetbi-luc and CaMKIIα-CreERT2/CAG-loxP.EGFP rats were processed with dual-label fluorescent IHC detecting EGFP and NeuN. Images were acquired using a confocal laser-scanning microscope. The ratio of GFP-positive/NeuN-positive neurons to all NeuN-positive neurons was calculated separately for cortex and hippocampal regions (CA1, CA3 and hilus). Mean and SEM were calculated from at least three rats per group.

### Commitment to distribute transgenic rat lines

All transgenic rat lines generated in this project will be made available upon request to scientists at academic institutions for non-commercial research or deposited into a repository/stock centre, making them available to the broader research community. We will process requests in an appropriate timely fashion and in the order of request received, provided the recipient will bear all costs for the shipment and sign our institutional Material Transfer Agreement. For scientists requesting rats harbouring components of the Tet system, the Notice and Acknowledgement Agreement (N&A) of 'TET Systems' available at http://www.tetsystems.com/ip-licensing/licensing/not-for-profit-research needs to be completed before these rats can be transferred to recipients.

## Abbreviations

ANOVA: analysis of variance; β-gal: β-galactosidase; CAG: chicken β-actin; Dox: doxycycline hydrochloride; E: embryonic day; EGFP: enhanced green fluorescent protein; GFP: green fluorescent protein; IHC: immunohistochemistry; P: postnatal day; PBS: phosphate-buffered saline; SEM: standard error of the mean; RLU: relative light units; tet: tetracycline; tTA: tetracycline-dependent transactivator.

## Competing interests

The authors declare that they have no competing interests.

## Authors' contributions

KS conceived the study, generated transgenic DNA constructs, collected and analysed experimental data and drafted the manuscript. TW participated in the design of the study, carried out the statistical analysis and drafted the manuscript. AF performed DNA microinjections and genotyping of the transgenic animals. LW functionally analysed the expression pattern of the CaMKIIα-tTA and CaMKIIα-CreERT2 founder lines by X-Gal stainings, luciferase measurements and IHC. BP helped in the cloning of transgenic constructs. DD performed *in vivo *luciferase imaging experiments. HB and DB conceived the study and helped to draft the manuscript. All authors read and approved the final manuscript.

## Supplementary Material

Additional file 1 **CreERT2 protein expression in the brain of transgenic CaMKIIα-CreERT2 rats**. CaMKIIα-CreERT2 rats were injected twice within 12 hours with tamoxifen for nuclear localization of the Cre recombinase. Three hours after the second injection, animals were prepared for analysis. Brain sections were immunostained using a Cre antibody. The most prominent Cre immunoreactivity was found in hippocampal pyramidal neurons (B,C,E,G) and in cortical structures. CaMKIIα-CreERT2 line 327 (A,B); line 396 (C,D); line 408 (E,F) and line 404 (G,H). Click here for file

Additional file 2 **Constitutive β-galactosidase expression in transgenic pCAG-loxP.EGFP Cre reporter rats**. Macroscopic appearance of X-Gal-stained tissues revealed strong b-galactosidase expression in the lung, kidney, muscle, heart, spleen and gastrointestinal tract including appendix and pancreas. Click here for file
