# Supplementary figures and images for: Conditional gene expression systems in the transgenic rat brain
Source: BMC Biol. 2012 Sep 3;10:77. doi: 10.1186/1741-7007-10-77 (PMC3520851; doi:10.1186/1741-7007-10-77)

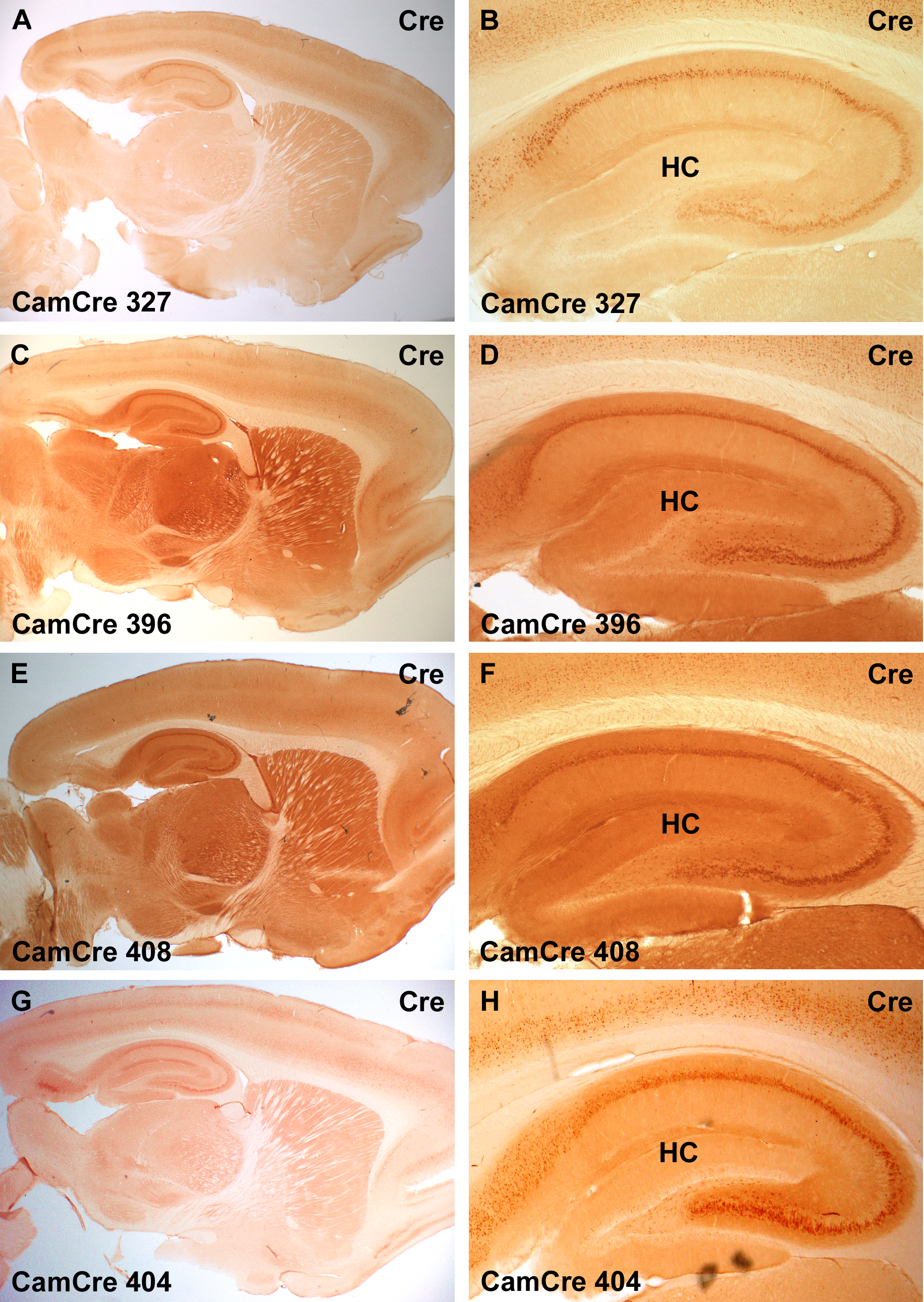

Supplement: Additional file 1 — CreERT2 protein expression in the brain of transgenic CaMKIIα-CreERT2 rats. CaMKIIα-CreERT2 rats were injected twice within 12 hours with tamoxifen for nuclear localization of the Cre recombinase. Three hours after the second injection, animals were prepared for analysis. Brain sections were immunostained using a Cre antibody. The most prominent Cre immunoreactivity was found in hippocampal pyramidal neurons (B,C,E,G) and in cortical structures. CaMKIIα-CreERT2 line 327 (A,B); line 396 (C,D); line 408 (E,F) and line 404 (G,H). [file 1741-7007-10-77-S1.TIFF]

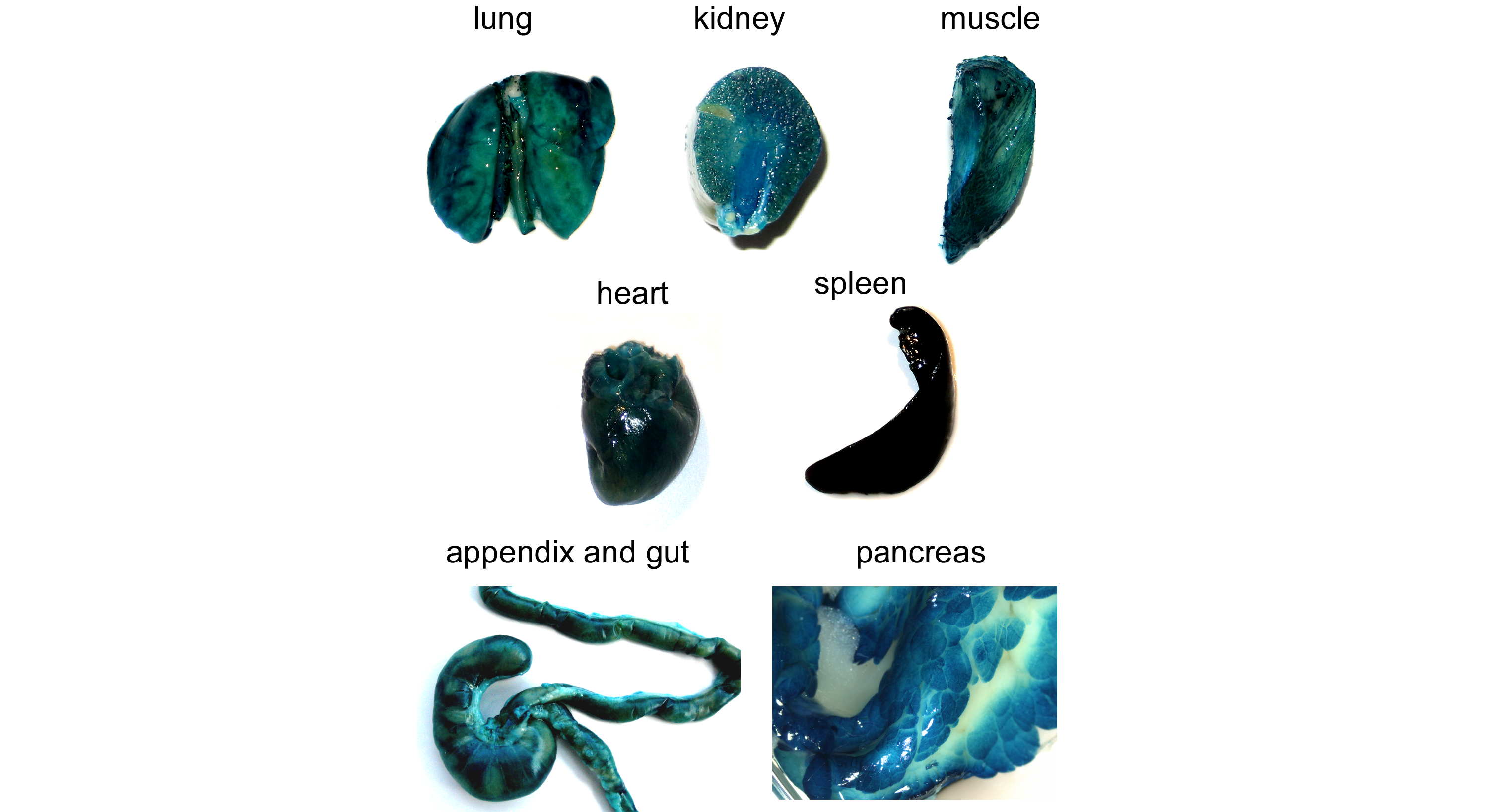

Supplement: Additional file 2 — Constitutive β-galactosidase expression in transgenic pCAG-loxP.EGFP Cre reporter rats. Macroscopic appearance of X-Gal-stained tissues revealed strong b-galactosidase expression in the lung, kidney, muscle, heart, spleen and gastrointestinal tract including appendix and pancreas. [file 1741-7007-10-77-S2.TIFF]
